# Distinct monocyte subset phenotypes in patients with different clinical forms of chronic Chagas disease and seronegative dilated cardiomyopathy

**DOI:** 10.1371/journal.pntd.0006887

**Published:** 2018-10-22

**Authors:** Damián E. Pérez-Mazliah, Melisa D. Castro Eiro, María Gabriela Álvarez, Bruno Lococo, Graciela Bertocchi, Gonzalo César, María A. Natale, María C. Albareda, Rodolfo Viotti, Susana A. Laucella

**Affiliations:** 1 Instituto Nacional de Parasitología Dr. Fatala Chaben, Buenos Aires, Argentina; 2 Hospital Interzonal General de Agudos Eva Perón, Buenos Aires, Argentina; Instituto de Investigaciones Biotecnológicas, ARGENTINA

## Abstract

**Background:**

Chronic infection with *Trypanosoma cruzi* leads to a constant stimulation of the host immune system. Monocytes, which are recruited in response to inflammatory signals, are divided into classical CD14^hi^CD16^—^, non-classical CD14^lo^CD16^+^ and intermediate CD14^hi^CD16^+^ subsets. In this study, we evaluated the frequencies of monocyte subsets in the different clinical stages of chronic Chagas disease in comparison with the monocyte profile of seronegative heart failure subjects and seronegative healthy controls. The effect of the anti-parasite drug therapy benznidazole on monocyte subsets was also explored.

**Methodology/Principal findings:**

The frequencies of the different monocyte subsets and their phenotypes were measured by flow cytometry. *Trypanosoma cruzi*-specific antibodies were quantified by conventional serological tests. *T*. *cruzi*-infected subjects with mild or no signs of cardiac disease and patients suffering from dilated cardiomyopathy unrelated to *T*. *cruzi* infection showed increased levels of non-classical CD14^lo^CD16^+^ monocytes compared with healthy controls. In contrast, the monocyte profile in *T*. *cruzi*-infected subjects with severe cardiomyopathy was skewed towards the classical and intermediate subsets. After benznidazole treatment, non-classical monocytes CD14^lo^CD16^+^ decreased while classical monocytes CD14^hi^CD16^—^increased.

**Conclusions/Significance:**

The different clinical stages of chronic Chagas disease display distinct monocyte profiles that are restored after anti-parasite drug therapy. *T*. *cruzi*-infected subjects with severe cardiac disease displayed a profile of monocytes subsets suggestive of a more pronounced inflammatory environment compared with subjects suffering from heart failure not related to *T*. *cruzi* infection, supporting that parasite persistence might also alter cell components of the innate immune system.

## Author summary

Monocytes are key players during infection, and they leave the bloodstream and migrate into tissues in response to inflammatory signals. Although the recruitment of monocytes is essential for the effective control and clearance of microorganisms, they can also be highly damaging to neighboring tissues. Based on the expression of CD14 and CD16, monocytes are classified into classical, non-classical and intermediate subsets, all of which exert different functions. Because chronic *T*. *cruzi* infection induces a constant activation of the host immune system, inflammatory signals are exacerbated, possibly leading to alterations in the frequencies of monocyte subsets. In this study, we evaluated the monocyte profile in *Trypanosoma cruzi*-infected subjects with different degrees of cardiac dysfunction and explored whether this profile was similar between seropositive and seronegative subjects with heart failure. We found that the different clinical stages of chronic Chagas disease displayed distinct monocyte profiles, which are susceptible to being restored by modulating the parasite load with anti-parasite drug therapy. *T*. *cruzi*-infected subjects with severe cardiac disease displayed a profile of monocytes subsets suggestive of a more pronounced inflammatory environment compared with subjects suffering from heart failure not related to *T*. *cruzi* infection, supporting that parasite persistence might be a detrimental factor in the evolution of the cardiac disease induced by *T*. *cruzi*.

## Introduction

Chagas disease, caused by infection with the intracellular protozoan parasite *Trypanosoma cruzi*, affects 6–7 million people and represents the most frequent cause of infectious cardiomyopathy in the world [1,2]. Three factors are likely associated with the development of the more severe clinical forms of the disease: parasite burden, the capacity of the host immune response to control parasites in specific tissues, and the effectiveness of the host immune response to control tissue damage.

In response to inflammatory signals, circulating monocytes leave the bloodstream and migrate into tissues, where following conditioning by local growth factors, pro-inflammatory cytokines and microbial products, they differentiate into macrophages or dendritic cells. Although the recruitment of monocytes is essential for the effective control and clearance of microorganisms, they can also be highly damaging to neighboring tissues [3]. Human monocytes are divided into subsets on the basis of surface CD14 and CD16 expression [4]. CD14^hi^CD16^−^monocytes “classical Mo”, which are also referred to as classical monocytes, are the most prevalent monocyte subset in human blood, and they show a high expression of the chemokine receptor CCR2. Classical monocytes can migrate to sites of injury and infection, where they differentiate into inflammatory macrophages [5]. The CD16^+^ monocyte population comprises two subsets: the non-classical CD14^lo^CD16^+^ “non-classical Mo” and the intermediate CD14^hi^CD16^+^ monocytes “intermediate Mo” [4,6]. Both subsets exhibit low and mild CCR2 expression [7]. Whereas non-classical monocytes are involved in the process of patrolling with potent anti-inflammatory function and wound healing, intermediate monocytes share some phenotypic and functional features of both classical and non-classical monocytes and mainly exert a pro-inflammatory role [7]. The two CD16^+^ subsets are shown to expand in many inflammatory conditions (e.g., cancer, sepsis and stroke) and infections such as HIV [8–11] and tuberculosis [12].

In the chronic phase of Chagas disease, T cell responses become exhausted over time, presumably due to the constant stimulation of the host immune system in this decades-long infection [13–15]. This constant stimulation of the host immune system is also evident by the expansion of CD14^+^CD16^+^HLA-DR^++^ monocytes [16], which shows that adaptive and innate immune responses can be disrupted in the chronic phase of the infection. Chronic Chagas heart disease presents morphological particularities that could account for a worsened clinical course compared with dilated cardiomyopathy not related to *T*. *cruzi* infection [17].

Herein, we sought to evaluate the frequencies of classical, intermediate and non-classical monocytes in the different clinical stages of chronic Chagas disease compared with the monocyte profile in seronegative dilated cardiomyopathy patients (DCM) and seronegative healthy controls. The effect of the anti-parasite drug therapy benznidazole on monocyte subsets was also explored in chronically infected subjects.

## Materials and methods

### Ethics statement

The protocol was approved by the institutional review boards of Hospital Interzonal General de Agudos Eva Perón, Buenos Aires, Argentina. Signed informed consent was obtained from all individuals before inclusion in the study.

### Study participants

*T*. *cruzi*-infected adult volunteers were recruited at the Chagas Disease Unit of Hospital Interzonal General de Agudos Eva Perón, Buenos Aires, Argentina. *T*. *cruzi* infection was determined by indirect immunofluorescence assay, hemagglutination assay, and enzyme-linked immunosorbent assay (ELISA) in compliance with domestic and international criteria [1]. The ELISA was carried out with a 1/200 dilution of the samples incubated in microplates precoated with *T*. *cruzi* epimastigote antigens. The binding of specific antibodies was detected with a horseradish peroxidase-labeled anti-human IgG antibody (Sigma). After addition of the substrate *o*-phenylenediamine (Sigma), the optical density at 490 nm (OD_490_) was quantified in an ELISA microplate reader (Model 550; Bio-Rad, Tokyo, Japan) [18]. The chronically infected seropositive subjects were clinically evaluated and stratified according to a modified version of the Kuschnir grading system [19,20]. The individuals in group 0 had normal electrocardiographic (ECG), normal chest radiographic, and normal echocardiographic findings; the subjects in group 1 had normal chest radiographic and echocardiographic findings but abnormal ECG findings; the subjects in group 2 had ECG abnormalities and heart enlargement; and the subjects in group 3 had ECG abnormalities, heart enlargement, and clinical or radiological evidence of heart failure. A group of seronegative subjects suffering from DCM with systolic heart failure were recruited for comparison of the monocyte subset phenotypes among patients with heart failure due to different disease etiologies. The inclusion criteria for patients with heart failure were class I/II/III (New York Heart Association classification), with an ejection fraction of <40% by echocardiography. The etiology for heart failure was hypertension in three patients, post-chemotherapy with doxorubicin in one patient who had no cancer at the time of study inclusion, alcoholism in one patient, and idiopathic DCM in three patients. Seronegative (uninfected) healthy controls were also included. Subjects with acute coronary syndrome, cancer, HIV, syphilis, diabetes, arthritis, or serious allergies at the time of study inclusion were excluded. At the time of the study, all of the participants were living in Buenos Aires, where *T*. *cruzi* infection is not endemic. After inclusion in the study, nine *T*. *cruzi*-infected subjects in the G0 group and three subjects in the G1 clinical group were treated with 5 mg·kg per day of benznidazole for 30 days [21,22]. Clinical, serological, and immunological analyses were performed prior to and at different time points after the treatment. Data on the number, sex, and age of the enlisted subjects are summarized in Table 1.

**Table 1:** Characteristics of the study population.

Study group	N	Age range ^A^ (median), years	Years of residence in endemic areas Median (range)	Gender	
				Male	Female
G0 ^B^	23	21–60 (43)^C^	18 (1–42)	8	15
G1 ^B^	6	24–58 (40)	13 (1–23)	5	1
G2-G3 ^B^^,^ ^D^	10	46–76 (54)^E^	20 (0–14)	8	2
Dilated cardiomyopathy ^F^	8	34–64 (59)	0	7	1
Uninfected healthy controls	17	21–58 (47)	0	8	9

Note.

^A^ Age at entry of the study.

^B^ Seropositive subjects were grouped according to the modified Kuschnir classification [19,20].

^C^ P < 0.05 compared with G2-G3 and dilated cardiomyopathy group by ANOVA followed by Bonferroni test.

^D^ Three and seven *T*. *cruzi*-infected subjects were classified as G2 and G3 patients, respectively.

^E^ P < 0.05 compared with uninfected healthy controls by ANOVA followed by Bonferroni test.

^F^ Seronegative subjects with heart failure and without an epidemiological background for *T*. *cruzi* infection.

### Collection of peripheral blood mononuclear cells (PBMCs) and serum specimens

Whole blood was drawn by venipuncture into heparinized tubes (Vacutainer; BD Biosciences). PBMCs were isolated by density gradient centrifugation on Ficoll-Hypaque (Amersham) and diluted in RPMI media containing 10% newborn bovine serum, 100 units/ml penicillin, 0.1 mg/ml Streptomycin, 2 mM L-glutamine and 10 mM HEPES buffer. The viability of the cells was checked by trypan blue staining with a viability range of 80–90%. A blood aliquot was allowed to coagulate at room temperature and centrifuged at 1000 g for 15 min for serum separation.

### Ex vivo flow cytometry for phenotype analysis

Immediately after collection, 1 × 10^6^ PBMCs were stained with different combinations of FITC-labeled anti-CD14, PE-labeled anti-CD16, APC-labeled anti-CD45RA and AF647-labeled anti-CCR2 (all from BD Pharmingen) at 4°C for 30 min. The cells were then fixed with 2% paraformaldehyde and stored at 4°C until acquisition. The cells were acquired with a BD FACS Calibur flow cytometer (BD Biosciences) and analyzed with FlowJo software v9.6 (Tree Star). Monocyte subsets were first selected on the basis of forward-scattered (FSC) vs. side-scattered (SSC) lights, and CD14^+^ cells were subsequently gated. From this population, CD14 vs. CD16 dot plots were drawn to establish the different CD14^+^ monocyte subsets (S1A, S1B and S1D Fig). For monocyte phenotyping, histograms for the expression of CD45RA and CCR2 were plotted for each monocyte subset (S1E Fig). Unstained and fluorescence minus one (FMO) samples were used as gating controls (S1C and S1E Fig). To demonstrate that the majority of the cells selected by the monocyte gating were truly monocytes, additional analyses were performed as follows. The PerCP-labeled anti-HLA-DR antibody (Biolegend) was added to the combination of CD14 and CD16 antibodies mentioned above, and CD14 vs. CD16 dot plots were drawn after the selection of HLA-DR^+^ cells from the CD14^+^-gated population [23,24] (S1A, S1B, S1E and S1F Fig). Staining with the APC-labeled anti-CD19, APC-Cy7-labeled anti-CD3, PE-labeled anti-CD14, and FITC-labeled anti-CD16 antibodies and with FV510 was performed to ascertain the contribution of any possible contaminating cells, including B, T, to the proportion of the different monocyte subsets. These additional assays were carried out on a FACS Aria II flow cytometer (BD Biosciences; S1 Fig).

### Statistics

The demographic and clinical characteristics of *T*. *cruzi*-infected subjects included in this study were summarized using the range and median. The normality of data was evaluated by the Shapiro-Wilk test. Differences among groups were evaluated by ANOVA followed by a Bonferroni/Dunn test for multiple comparisons or the Kruskal-Wallis test followed by post-tests, as appropriate. To evaluate the changes in monocyte subsets over time post-treatment compared with the baseline, a linear mixed model with compound symmetry and time as a fixed effect was used to maximize the utilizable data, as some subjects had missing data. The correlation between the frequencies of monocyte subsets and the post-treatment/pretreatment ratio of *T*. *cruzi*-specific antibodies measured by ELISA assay was determined by Spearman’s test. The networks comprising the different monocyte subsets were created after performing a correlation analysis by Spearman’s correlation test. Differences were considered statistically significant at P <0.05.

## Results

### Perturbations of monocyte phenotypes in patients with chronic Chagas disease and uninfected subjects with dilated cardiomyopathy

**Fig 1:**
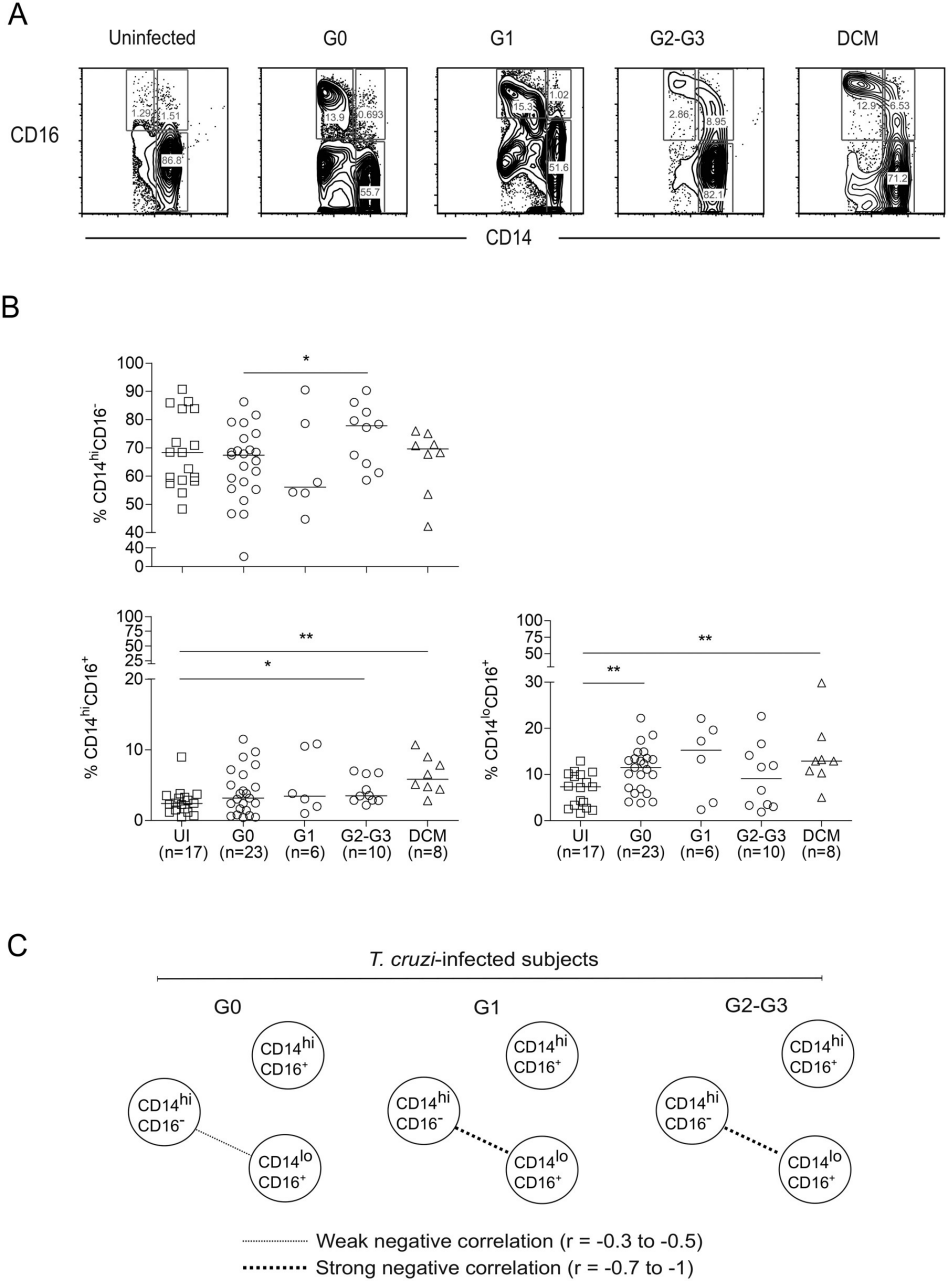
Measurement of monocyte subsets in chronic Chagas disease patients and seronegative subjects with dilated cardiomyopathy. (A) Representative plots for single subjects of the different clinical groups (i.e., G0, G1 and G2-G3 patient groups among untreated *T*. *cruzi*-infected subjects; seronegative subjects with dilated cardiomyopathy (DCM) and uninfected healthy controls) showing CD14 and CD16 expression. Monocytes were selected based on forward (FSC) and side scattering (SSC), and the different CD14^+^ monocyte subsets were analyzed by the expression of CD14 and CD16 using flow cytometry. (B) Each point represents the frequency of different monocyte subsets for individual subjects. The median values are indicated by the horizontal lines. * P < 0.05; ** P < 0.01; *** P < 0.001. Differences among groups were evaluated by ANOVA or the Kruskal-Wallis test followed by post-tests. (C) Classical, intermediate and non-classical monocyte subsets are depicted by the corresponding circles; each connecting line represents a significant interaction (P < 0.05) determined by Spearman’s correlation test.

On the basis of the CD14 and CD16 expression levels, CD14^+^ monocytes were subdivided into classical (CD14^hi^CD16^−^, “classical Mo”), intermediate (CD14^hi^CD16^+^, “intermediate Mo”), and non-classical (CD14^lo^CD16^+^, “non-classical Mo”) subsets (Fig 1A and S1 Fig) and were quantified in untreated *T*. *cruzi*-infected subjects, seronegative (uninfected) patients with DCM, and in seronegative healthy controls. The frequencies of the different monocyte subsets did not change significantly after preselection of HLA-DR^+^ cells from the CD14^+^-gated population (S1A, S1B, S1E and S1F Fig). The preselection of HLA-DR^+^ cells allowed for the exclusion of HLA-DR–negative NK cells [23,24]. We confirmed that the contribution of CD3^+^ and CD19^+^ cells to the frequencies of the different monocyte subsets selected from the total CD14^+^-gated population was very low either in *T*. *cruzi*-infected or uninfected subjects. As presented in S2 Fig, 1.58% of all CD14^+^ monocytes in an uninfected subject showed positive staining for CD19; this figure represents the final frequency of 1.3%, 0.12%, and 0.094% of classical, intermediate, and non-classical monocytes, respectively. Likewise, 2.54% of all CD14^+^ monocytes stained for CD3; this percentage represents the final frequency of 1.84%, 0.1%, and 0.072% of classical, intermediate, and non-classical monocytes, respectively.

The frequency of classical monocytes was higher in *T*. *cruzi*-infected subjects with severe cardiomyopathy (i.e., the G2 and G3 clinical groups) than in patients with no signs of cardiac disease (i.e., the G0 clinical group). Although not statistically significant, patients in groups G2 and G3 had higher frequencies of the CD14^hi^CD16^–^ “classical Mo” monocyte subset than those of the uninfected healthy controls and G1 and DCM patients (Fig 1B). In contrast, *T*. *cruzi*-infected subjects with severe cardiomyopathy had similar frequencies of non-classical CD14^lo^CD16^+^ monocytes to the uninfected healthy control levels, whereas *T*. *cruzi*-infected subjects in the G0 clinical group and patients suffering from DCM unrelated to *T*. *cruzi* infection (DCM) showed significantly higher levels of non-classical CD14^lo^CD16^+^ monocytes than the uninfected healthy controls (Fig 1B). A slight increase in non-classical CD14^lo^CD16^+^ monocytes in G1 patients compared with that in the uninfected healthy controls was also observed. Increased frequencies of intermediate CD14^hi^CD16^+^ monocytes were found in chronically infected subjects with more severe stages of the disease and DCM compared with the levels in uninfected healthy controls. The CD14^hi^CD16^+^ “intermediate Mo” frequencies were also slightly (but not significantly) increased in patients with less severe forms of Chagas disease (Fig 1B). Although patients with chronic Chagas disease with heart failure were older than those in the G0 and the uninfected healthy control groups, we did not find any correlation between the age of the subjects and the frequency of the different monocyte subsets in our study cohort (classical Mo r = 0.219, P = 0.518; intermediate Mo r = –0.002, P = 0.996; non-classical Mo r = 0.033, P = 0.923;). A distinct network profile of monocyte subsets was observed in *T*. *cruzi*-infected subjects that varied according to disease severity. The G0 group, with no signs of cardiac disease, had a moderate inverse correlation between classical CD14^hi^CD16^–^ and non-classical CD14^lo^CD16^+^ monocyte subsets (Fig 1C). This correlation was not observed in the uninfected healthy controls (r = -0.329, P = 0.198) or seronegative subjects with DCM (r = -0.359, P = 0.389). The inverse correlation between classical and non-classical monocyte subsets was increased in the G1 patient group and was sustained in patients with Chagas disease with more severe cardiomyopathy (Fig 1C).

### Phenotyping of the different monocyte subsets

**Fig 2:**
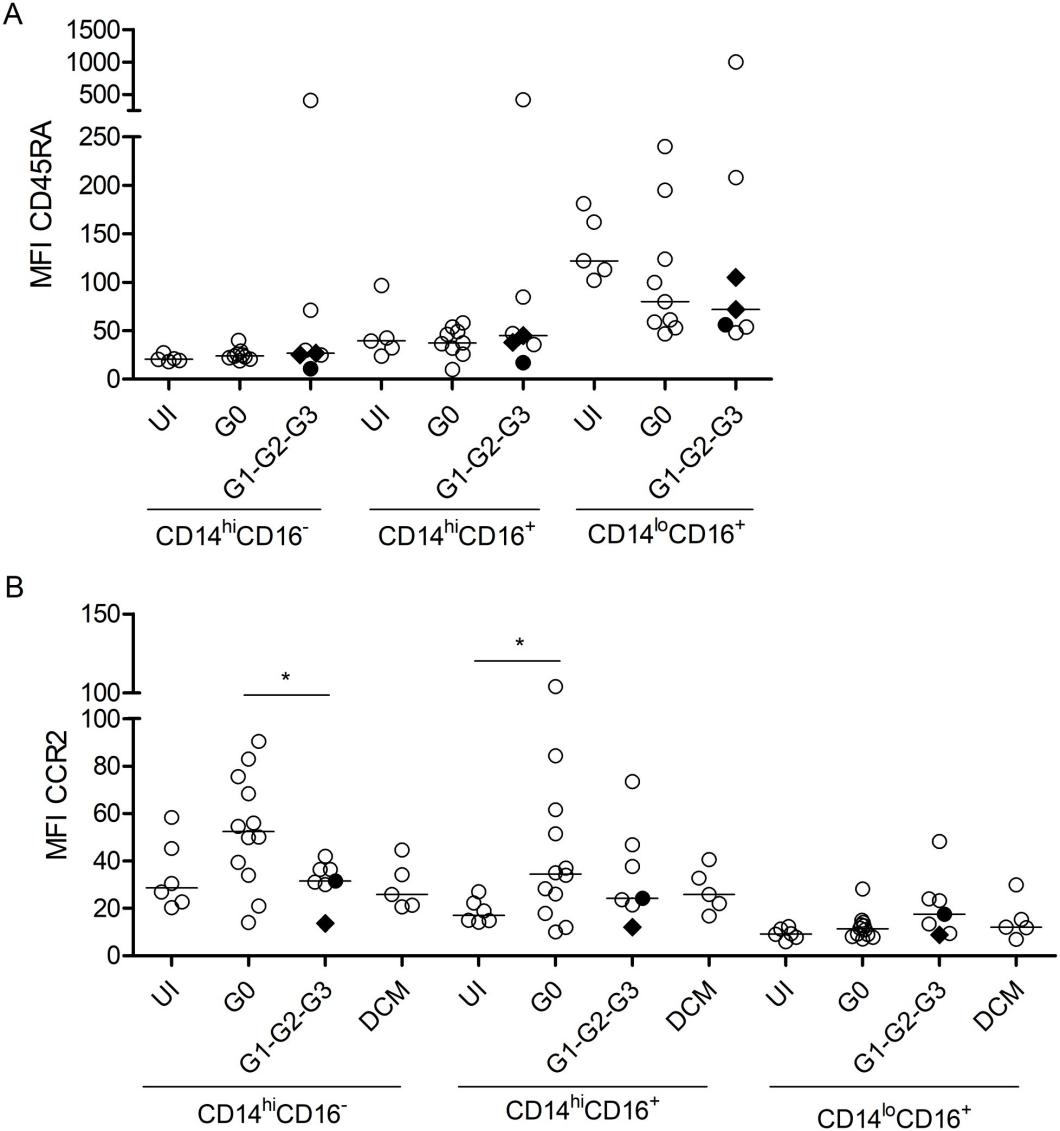
Phenotyping of the different monocyte subsets. PBMCs were stained for CD14, CD16, CCR2, HLA-DR and CD45RA and analyzed by flow cytometry. Monocytes were gated by forward and side scattering. Each point represents the MFI for CD45RA (A) and CCR2 (B) in the different monocytes subsets among *T*.*cruzi*-infected subjects (G0 n = 21; G1-G2-G3 n = 7), seronegative dilated cardiomyopathy patients (DCM) (n = 5) and uninfected healthy controls (n = 6). Black diamonds indicate G1 benznidazole-treated patients; the black circle indicates a G2 benznidazole-treated patient. The median values are indicated by the horizontal lines. * P < 0.05; Differences among groups were evaluated by ANOVA, the Kruskal-Wallis test followed by post-tests or a *t*-test.

The expression of CD45RA in the different monocyte subsets concurred with the expression data reported in other studies [25–27], and did not vary between patients with chronic Chagas disease regardless the clinical status and uninfected healthy controls (Fig 2A and S1 Fig). In contrast, chronically infected subjects with no signs of cardiac dysfunction had CD14^hi^CD16^–^ and CD14^hi^ CD16^+^ monocyte subsets with higher CCR2 expression than those found in patients with severe cardiac disease and in the uninfected healthy controls, respectively (Fig 2B and S1 Fig).

### Monitoring of monocyte subsets after treatment with benznidazole

**Fig 3:**
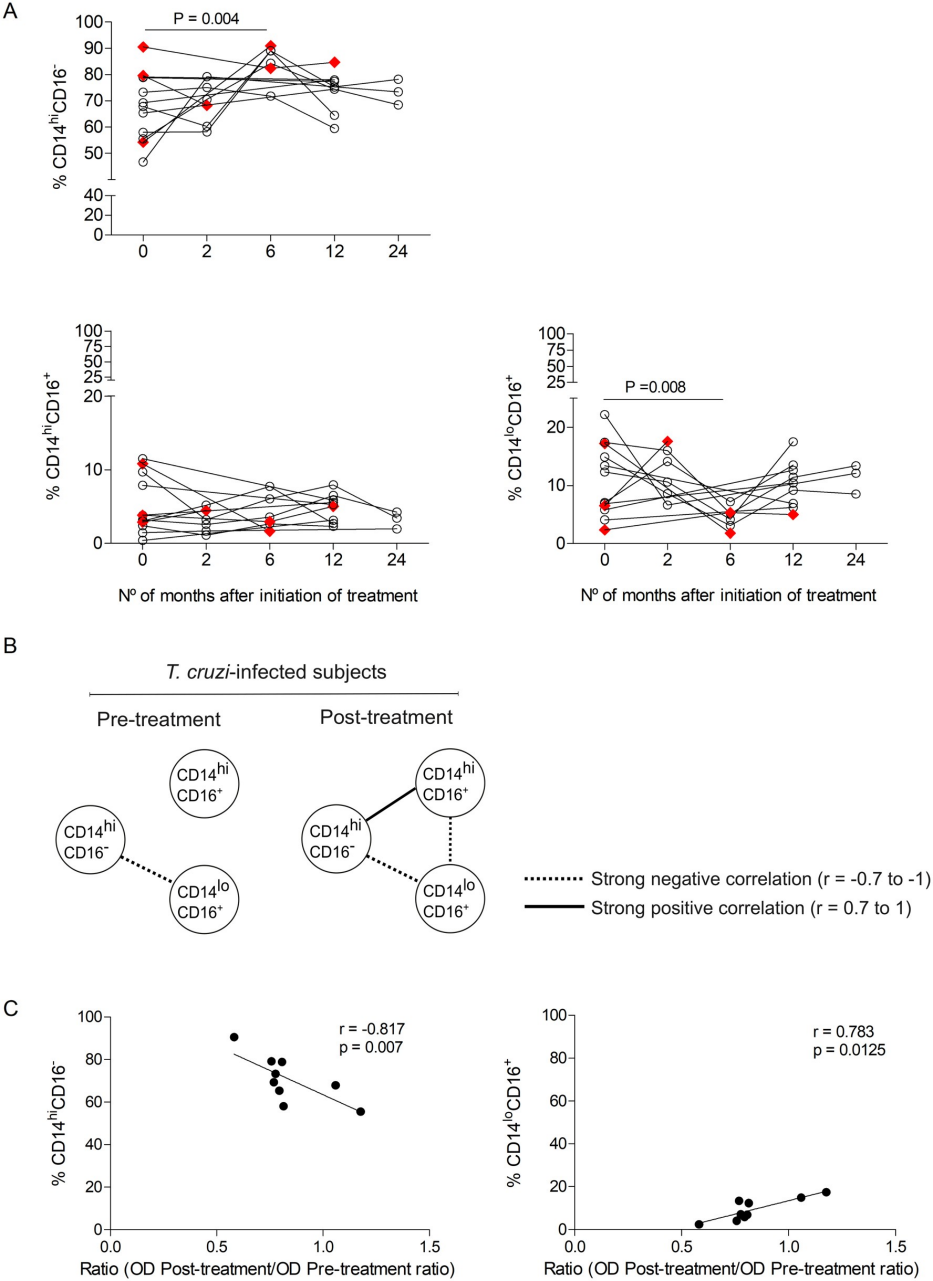
Monitoring of monocyte subsets after drug therapy in subjects chronically infected with *Trypanosoma cruzi*. (A) Monocyte subsets were measured prior to and after therapy with benznidazole in 12 *T*. *cruzi*-infected subjects (G0, black circles; G1, red diamonds). Changes in the monocyte subsets post-treatment relative to baseline were analyzed by a Linear Mixed Model. (B) Classical, intermediate, and non-classical monocyte subsets prior to and after treatment with benznidazole are depicted by the corresponding circles. Each connecting line represents a significant interaction (P < 0.05) determined by Spearman’s correlation test. (C) Correlation analysis between the baseline frequencies of classical or non-classical monocyte subsets and the ratio of post-treatment/pre-treatment *T*. *cruzi*-specific antibodies measured by ELISA at 12–24 months after treatment with benznidazole. Spearman’s correlation test between the frequency of CD14^hi^CD16^–^ “classical Mo” and CD14^lo^CD16^+^ “non-classical Mo” and the post-treatment/pretreatment ratio of the optical density obtained by the ELISA technique.

To address the relationship between the different monocyte subsets and parasite persistence, classical, non-classical and intermediate monocytes were measured in chronic Chagas disease patients prior to and following treatment with benznidazole. A sharp decrease in non-classical monocytes CD14^lo^CD16^+^ along with an increase in classical monocytes CD14^hi^CD16^-^ was observed six months after drug therapy (Fig 3A). Of note, the decrease in non-classical monocytes post-treatment was restricted to those patients who had baseline levels above the median values (i.e., non-classical Mo 1 patient group), whereas classical monocytes were increased post-treatment in patients who had baseline CD14^hi^CD16^-^ “classical Mo” frequencies under the median values (i.e., classical Mo 2 patient group) (Table 2). Likewise, when patients were classified by those who had baseline frequencies of intermediate CD14^hi^CD16^+^ monocytes above (i.e., intermediate Mo 1 patient group) or under (i.e., intermediate Mo 2 patient group) the median values, the frequencies of CD14^hi^CD16^+^ “intermediate Mo” decreased in the former group, while the other group presented no changes in this monocyte subset after benznidazole therapy (Table 2). Although the changes were more pronounced at six months post-treatment, the frequencies of classical and non-classical monocytes were in the range of the uninfected healthy controls (i.e., classical monocytes, range = 48.4%-90.7% and non classical monocytes, range = 1.56%-12.9%) by 12–24 months following drug therapy. Treatment with benznidazole changed the network profile, inducing a positive correlation between classical and intermediate monocyte subsets and a strong negative correlation between intermediate and non-classical monocyte subsets within 12–24 months of follow-up post-treatment (Fig 3B). We then evaluated whether the treatment efficacy, determined by the presence of significant decreases in *T*. *cruzi*-specific antibodies [20], was associated with a particular profile of monocyte subsets prior to drug therapy. An inverse correlation was observed between the frequencies of classical monocytes prior to treatment and the rate of decreases in *T*. *cruzi*-specific antibodies post-treatment (i.e., a lower ratio post-treatment vs. pre-treatment in subjects with high baseline frequencies of classical monocytes) (Fig 3C, S3 Fig). In contrast, the frequencies of non-classical monocytes prior to treatment were positively correlated with the decrease in parasite-specific antibodies post-treatment (Fig 3C) (i.e., a lower ratio post-treatment vs. pre-treatment in subjects with low baseline frequencies of non-classical monocytes).

**Table 2:** Mixed model analysis of non-classical and intermediate monocyte subsets in chronic Chagas disease patients following therapy with benznidazole according to baseline frequencies.

Patient group	Dependent variable	No. of months Post-treatment	Estimate ^A^	95% CI	*P*-value
Classical Mo 1	CD14^hi^CD16^—^ ^B^				
(n = 6)		Month 2	−4.84	−18.95, 9.25	0.464
		Month 6	−1.14	−15.24, 12.96	0.861
		Month 12	−3.13	−13.65, 7.38	0.525
Classical Mo 2	CD14^hi^CD16^—^ ^C^				
(n = 6)		Month 2	8.63	0.81, 18.07	0.071
		Month 6	29.24	19.81, 38.68	< 0.001
		Month 12	13.05	3.61, 22.48	0.010
		Month 24	16.96	6.59, 27.34	0.004
Intermediate Mo 1	CD14^hi^CD16^+^ ^B^				
(n = 6)		Month 2	−10.36	−15.81, −4.93	0.001
		Month 6	−15.57	−20.99, -10.14	< 0.001
		Month 12	−6.04	−11.33, −0.76	0.028
		Month 24	−8.43	−14.08, −2.77	0.006
Intermediate Mo 2	CD14^hi^CD16^+^ ^C^				
(n = 6)		Month 2	0.38	−1.60, 2.36	0.69
		Month 6	1.61	−0.52, 3.73	0.13
		Month 12	1.56	−0.32, 3.44	0.098
		Month 24	0.53	−2.20, 3.26	0.69
Non-classical Mo 1	CD14^lo^CD16^+^ ^B^				
(n = 6)		Month 2	−6.22	−10.94, −1.52	0.013
		Month 6	−13.18	−17.87, −8.48	< 0.001
		Month 12	−5.26	−9.95, −0.56	0.031
		Month 24	−4.94	−10.01, 0.14	0.050
Non-classical Mo 2	CD14^lo^CD16^+^ ^C^				
(n = 6)		Month 2	10.46	5.52, -15,40	0.001
		Month 6	0.77	−4.16, 5.71	0.737
		Month 12	4.07	0.41, 7.74	0.032

Note.

^A^ The estimate value for each time point post-treatment obtained by linear mixed model analysis indicates an approximation of the fold-change compared with the baseline. Negative values denote a decrease in the frequency of the indicated monocyte subset while positive values denote an increase in the frequency of the indicated monocyte subset compared with baseline;

^B^ Subjects with frequencies of the indicated monocyte subset above median values (i.e., patient group 1);

^C^ Subjects with frequencies of the indicated monocyte subset under median values (i.e., patient group 2).

## Discussion

Although the cause of morbidity in chronic *T*. *cruzi* infection has been the source of much debate and controversy, most of the available data support the conclusion that Chagas disease is the result of the failure of the immune system to completely clear this persistent infection and the resulting effects of decades of immune assault [28,29].

In the present study, we found that the monocyte profile in chronically *T*. *cruzi*-infected subjects varies according to disease severity and changes after anti-*T*. *cruzi* treatment with benznidazole. The monocyte profile in less severe forms of cardiac disease is enriched in non-classical monocytes (CD14^lo^CD16^+^), whereas the monocyte profile in Chagas disease patients with severe cardiomyopathy is skewed toward classical and intermediate monocytes. These findings suggest that patients without signs of cardiac dysfunction or mild cardiac disease have a more balanced monocyte profile with both proinflammatory and anti-inflammatory tissue repair capacity [5]. Recent studies revealed that classical monocytes exit from bone marrow into the blood stream, where they give rise to intermediate monocytes, which subsequently differentiate into non-classical monocytes [11,30]. Other authors have reported that non-classical monocytes may also arise independently from myeloid progenitors in the bone marrow [31]. The inverse correlation between classical and non-classical monocytes in chronically *T*. *cruzi*-infected subjects suggests that non-classical monocytes may derive from classical monocytes. Nonetheless, this inverse association may also be due to more active recruitment of classical than non-classical monocytes in *T*. *cruzi*-infected tissues in the G0 and G1 groups. In line with these findings, the enhanced expression of CCR2 in classical and intermediate monocytes of *T*. *cruzi*-infected patients without signs of cardiac disease may support more active recruitment of these monocyte subsets.

Upon activation, classical monocytes produce inflammatory cytokines, may exert phagocytic and myeloperoxidase activities, and release heightened levels of superoxide [32]; these actions altogether can help to keep the parasite under control. Although these responses maintained over time may also result in tissue damage, the recruitment of non-classical monocytes may counteract these harmful effects. In contrast, a more inflammatory environment and tissue damage observed in patients with severe stages of chronic Chagas disease may be responsible for more active chemoattraction and recruitment of non-classical monocytes to sites of inflammation to perform their anti-inflammatory and tissue repair functions [5], accounting for the stronger inverse correlation between classical and non-classical monocytes in these patients. The extensive fibrosis observed in Chagas disease patients with heart failure might be a consequence of exacerbated remodeling [33] mediated by the tissue repair function of non-classical monocytes. Of note, no correlation between classical and non-classical monocytes was observed in DCM, in agreement with the low-grade inflammation associated with heart failure of non-infectious origin [34]. Several studies suggest that *T*. *cruzi*-infected subjects with an indeterminate form of the chronic disease have an overall modulatory cytokine profile of monocytes, with the production of IL-10 as a counterbalance cytokine [16,35–40]. In line with our findings, a recent study revealed that chronically *T*. *cruzi*-infected subjects with cardiac dysfunction have an increased frequency of intermediate monocytes [41]. Nevertheless, the increased counts of both non-classical monocytes at less severe clinical stages in our study are at odds with the results reported by other authors [40,41].

Treatment with benznidazole induced reductions in both non-classical and intermediate monocyte subsets along with an increase in classical-monocyte numbers. This finding is probably due to a reduction in parasite load and the subsequent decrease in inflammation and recruitment of non-classical and intermediate monocytes, thus enabling replenishment of classical monocyte subsets in the circulation. The inverse correlation between non-classical and intermediate monocytes after benznidazole therapy suggests that the signals that induce the rise in the number of non-classical monocytes in the G0 and G1 clinical groups, which are the target populations for trypanocidal treatment, may disappear after this therapy. It has been shown that monocytes can be reprogrammed and switch from a wound-healing to a pro-inflammatory state in response to changes in inflammatory stimuli [42,43]. Accordingly, we recently observed that the levels of MCP-1, one of the main chemokines that regulate migration and infiltration of monocytes and/or macrophages, and of IP-10, which acts as a modulator of angiogenesis and wound healing, decrease in *T*. *cruzi*-infected children after treatment with benznidazole (Albareda MC, personal communication). In contrast to our findings, Sathler-Avelar et al. demonstrated that a pediatric population of *T*. *cruzi*-infected subjects treated with benznidazole has higher percentages of non-classical CD14^+^CD16^+^ cells as compared with an untreated group [44]. These two studies have several distinct features that might explain these apparent discrepancies, including the difference in the classification of monocytes and in the length of infection. Benznidazole treatment in the indeterminate phase of *T*. *cruzi* infection has been reported to downregulate monocyte phagocytic capacity [45], further supporting the overall low immune activation after benznidazole therapy. An important observation of our work is that the decline in *T*. *cruzi*-specific antibody levels after benznidazole therapy, suggestive of successful treatment, was associated with lower baseline levels of non-classical monocytes and higher baseline levels of classical monocytes. A proper balance among the different monocyte subsets may be critical for preventing persistent inflammation and for achieving controlled repair, which might also be an important factor for treatment efficacy.

One limitation of this study is that the functional status of the different monocyte subsets was not assessed prior to and after therapy. Additionally, the gating strategy employed did not allow us to exclude the low frequencies of contaminating B and T cells in the different CD14^+^ monocyte subsets.

In summary, *T*. *cruzi*-infected subjects with severe cardiac disease have a profile of monocyte subpopulations that is suggestive of a more pronounced inflammatory environment as compared with the subjects without signs of cardiac dysfunction and those with heart failure not related to *T*. *cruzi* infection. These findings further indicate that parasite persistence may also alter cell components of the innate immune system.

## Supporting information

S1 Fig Gating strategy. Monocytes were selected on the basis of forward (FSC) and side (SSC) scatter of light. CD14^+^ cells were subsequently selected and analyzed for the different monocyte subsets according to the expression of CD14 and CD16 (A-C). Alternatively, a gate on HLA-DR^+^ cells was drawn and the different monocyte subsets were analyzed (A, B, E and F). Classical, intermediate, and non-classical monocytes are gated in green, blue, and red, respectively. For each subset, the expression of CCR2, and CD45RA was analyzed with histogram plots (D). Unstained and fluorescence minus one (FMO) controls were used to determine the nonspecific antibody binding (D, E).
(TIF)

S2 Fig Multiparametric flow cytometry analysis for identification of truly monocytes. Monocytes were selected on the basis of forward (FSC) and side (SSC) scattering (A). Viable cells were gated by their negative staining for the viability marker FV510 (B) and single cells were gated based on FSC-W and FSC-A parameters (C). CD14^+^ cells were subsequently selected (D) and the different monocyte subsets were drawn according to the expression of CD14 and CD16 (E). Alternatively CD19^+^ (F-G) or CD3^+^ (H-I) cells were selected from the CD14^+^ gate and the different monocyte subsets were drawn as shown in E. The percentages indicate the frequencies of each monocyte subset out of total CD14^+^ (E), CD14^+^CD19^+^ (G) and CD14^+^CD3^+^ (I) cells in an uninfected subject.
(TIF)

S3 Fig Monitoring of *Trypanosoma cruzi*-specific antibodies by conventional serological tests following treatment with benznidazole. *T*. *cruzi*-specific antibodies, as determined by ELISA (A), hemagglutination assay (B), and immunofluorescence assays (C), were measured prior to and at different time points after the treatment with benznidazole. Each open circle represents the data for single subjects. Broken horizontal lines show the threshold of reactivity for each serological test. * P < 0.05 versus OD values prior to the treatment, by paired Student’s *t-*test.
(TIF)
