# Supplementary figures and images for: Distinct monocyte subset phenotypes in patients with different clinical forms of chronic Chagas disease and seronegative dilated cardiomyopathy
Source: PLoS Negl Trop Dis. 2018 Oct 22;12(10):e0006887. doi: 10.1371/journal.pntd.0006887 (PMC6211766; doi:10.1371/journal.pntd.0006887)

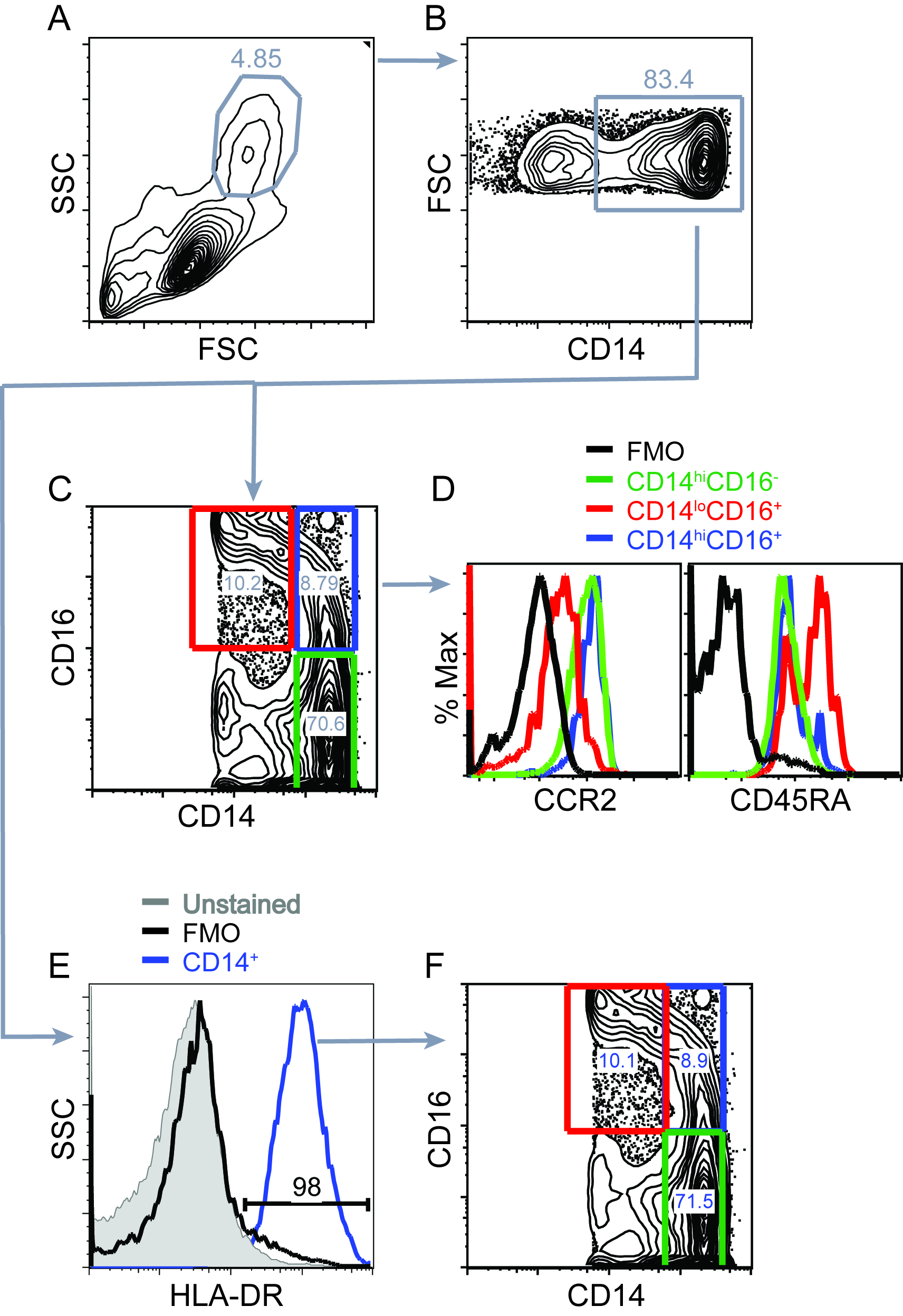

Supplement: S1 Fig — Monocytes were selected on the basis of forward (FSC) and side (SSC) scatter of light. CD14+ cells were subsequently selected and analyzed for the different monocyte subsets according to the expression of CD14 and CD16 (A-C). Alternatively, a gate on HLA-DR+ cells was drawn and the different monocyte subsets were analyzed (A, B, E and F). Classical, intermediate, and non-classical monocytes are gated in green, blue, and red, respectively. For each subset, the expression of CCR2, and CD45RA was analyzed with histogram plots (D). Unstained and fluorescence minus one (FMO) controls were used to determine the nonspecific antibody binding (D, E). (TIF) [file pntd.0006887.s001.tif]

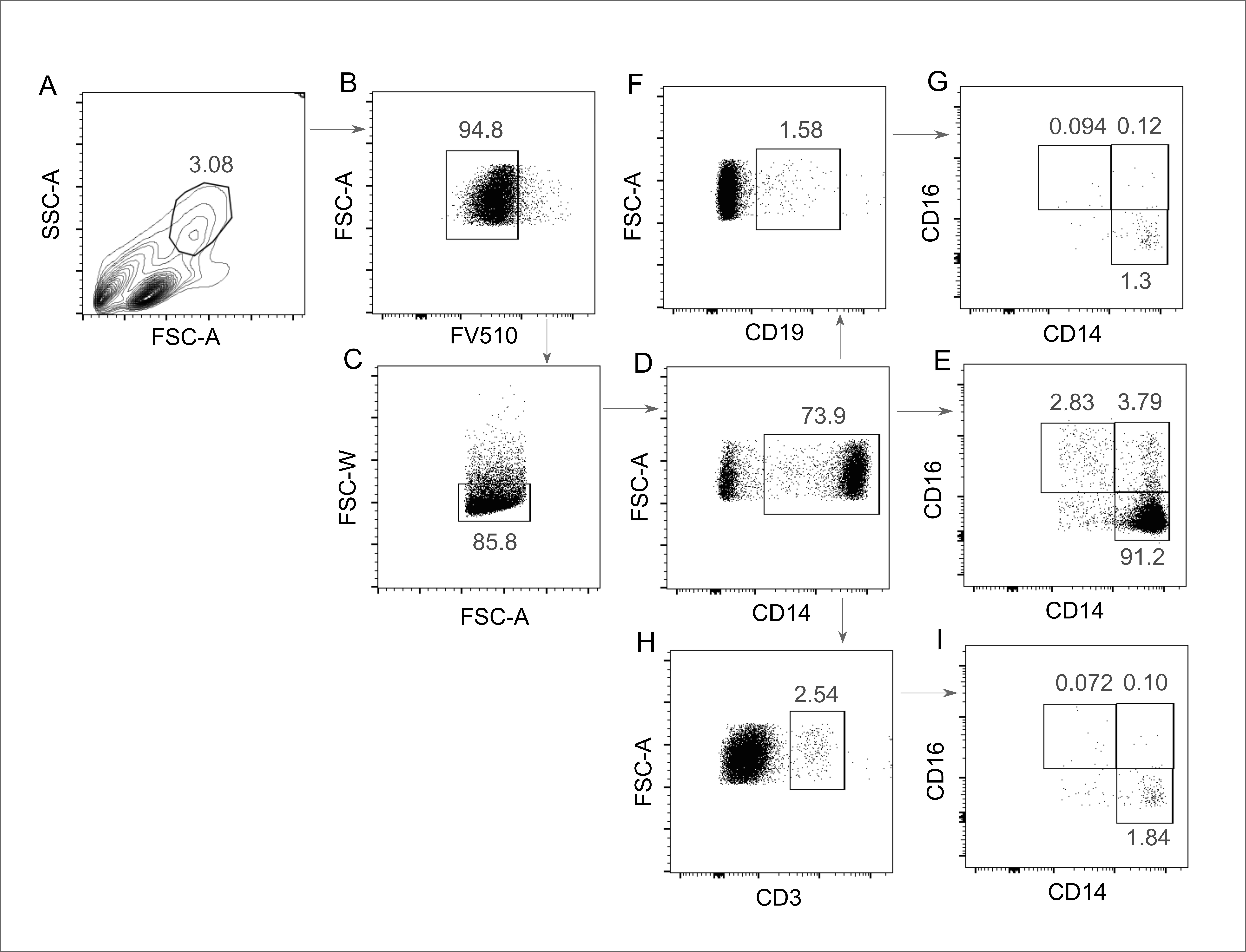

Supplement: S2 Fig — Monocytes were selected on the basis of forward (FSC) and side (SSC) scattering (A). Viable cells were gated by their negative staining for the viability marker FV510 (B) and single cells were gated based on FSC-W and FSC-A parameters (C). CD14+ cells were subsequently selected (D) and the different monocyte subsets were drawn according to the expression of CD14 and CD16 (E). Alternatively CD19+ (F-G) or CD3+ (H-I) cells were selected from the CD14+ gate and the different monocyte subsets were drawn as shown in E. The percentages indicate the frequencies of each monocyte subset out of total CD14+ (E), CD14+CD19+ (G) and CD14+CD3+ (I) cells in an uninfected subject. (TIF) [file pntd.0006887.s002.tif]
